# Comparative Cephalometric Study Between Nasal and Predominantly Mouth Breathers

**DOI:** 10.1016/S1808-8694(15)30037-9

**Published:** 2015-10-19

**Authors:** Jussara Marinho Dias Frasson, Maria Beatriz Borges de Araújo Magnani, Darcy Flávio Nouer, Vânia Célia Vieira de Siqueira, Nádia Lunardi

**Affiliations:** a Specialist in odontopediatrics, orthodontics and facial orthopedics. MSc in orthodontics. PhD student in physiology. Odontopediatrician, orthodontist and Guest Professor in Orthodontics.; b MS-3 Assistant Professor. Sub-coordinator of Orthodontics - FOP-Unicamp.; c Full professor of Orthodontics - FOP-Unicamp. Post graduation coordinator of Masters and Doctorate courses in Orthodontics.; d Assistant Professor, PhD – Post-Graduation Course of Orthodontics.; e MSc in Orthodontics. Guest Professor. FOP-UNICAMP.

**Keywords:** Nasorespiratory Function, Dentofacial Morphology, Orthodontics

## Abstract

**Aim:**

to evaluate the possible correlation between the respiratory pattern in determining the craniofacial dimensions, using as baseline the Tweed-Merrifield's cephalometric analysis, added to angle SN-GoGn and to Y axis angle.

**Methodology:**

The selected sample to this study comprised 50 teleradiographies taken in lateral and natural positions of the head in young female patients at the age of 9 to 12 years, presenting mean age of 10 years and 5 months and Class 1 malocclusion. After diagnosis of respiratory pattern, the sample was divided into two groups: control group, 25 teleradiographies of nasal breathers in lateral and natural positions of the head; experimental group, 25 teleradiographies of predominantly mouth breathers in lateral and natural positions of the head.

**Results:**

The results were submitted to descriptive analysis (mean and standard deviation), test F and “t” Student test with significance level of 5%. There was no significant difference between the group with nasal breathing and the group with predominantly mouth breathing for any of the studied variables.

## INTRODUCTION

The relationship between respiratory function with occlusal and craniofacial morphology development represents a long and controverted history, as far as orthodontics is concerned. Concerns about possible skeletal and dental alterations accruing from respiratory patterns have poked investigators for some time now in the areas of orthodontics, otolaryngology, speech therapy and others. Cheng et al. (1988), Cooper (1989)^1^, Parolo & Bianchini (2000), Queluz & Gimenez (2000)^2^, concluded that nasal obstruction multidisciplinary approach by otolaryngologists and orthodontic specialists, is of great advantage so as to reduce adverse effects caused by alterations in respiratory mode, occlusion and craniofacial morphology.

Oral breathing has a multifactorial etiology that may vary from an anatomical predisposition (narrow airway) such as physical obstructions – tonsil hypertrophy, adenoid hypertrophy, nasal polyps, nasal septum deviations, respiratory allergies, climatic conditions, sinusitis, turbinate hypertrophy, sleeping position, artificial breastfeeding, or from deleterious oral habits such as thumb sucking or pacifiers that depending on the intensity, frequence and habit duration may deform the dental arch and alter facial harmony (Andrade & Majolo, 2000^3^; Rodrigues & Rodrigues, 2003). Mocellin (1992)^4^ evaluated the relationship between oral breathing and dento-facial development, calling attention to the first 10 years of life when we have the most facial development. The author states that all patients with chronic nasal obstruction may become an oral breather, and the most frequent type of obstruction is adenoid hypertrophy.

Authors like: Linder-Aronson & Bäckström (1960), Ricketts (1968), Hawkins (1969)5, Paul & Nanda (1973), Carbone & Bernaba (1977), Linder-Aronson (1979)6, McNamara Jr. (1981)^7^, Bresolin et al. (1983)^8^, Melsen et al. (1987)^9^, Martinez Esteinou & Omana Vidal (1988), Cheng et al. (1988), Martins (1988), Yamada et al. (1997)^10^, Fujiki & Rossato (1999)^11^, Sabatoski (1999)^12^, Bizetto (2000)^13^, Motonaga et al. (2000)^14^, Mello (2001), Pereira et al. (2001), Simas Netta et al. (2004)^15^, have found a direct relationship between airway obstruction, persistence of such obstruction and development of the cranio-facial complex. It is believed that long standing obstruction causes oral breathing, which has a negative impact on the cranio-facial complex, leading to a set of functional dento-alveolar and skeletal changes in individuals, called “the long face syndrome”. Such syndrome is clinically characterized by postural open bite, narrow and underdeveloped nostrils, shorter upper lip, vestibular-version of upper incisive teeth, everted lower lip, vague facial expression, narrow and v-shaped maxillary arch, deep palate and class II malocclusions, cross bite, hypertrophic tonsils and adenoids, anterior open bite and lingual interposition (Ricketts, 1968).

Those who have a deviation from normal nasal breathing are considered mouth breathers, but this is often times insufficient; being, therefore, replaced by mouth supplement or mixed breathing (Lusvarghi, 1999)^16^.

According to Bueno Jr. (1996), total nose exclusion in breathing, in the long run leads to deep mucosal alterations. Mechanical obstruction inside the nose, as it happens in nasal septum deviations, turbinate hypertrophy or in scar tissue stenosis, may cause mouth breathing and its consequences, and also, may cause nasal and paranasal sinuses mucosal diseases.

The most common cause of mouth breathing is, without a doubt, nasal obstruction, this being even more important when we consider children, because they are growing and developing. One can never breath exclusively by mouth; what we have is dominant mouth breathing or mixed breathing (Lusvarghi, 1999)^16^. Nasal obstruction may be divided as to side (uni or bilateral) and as to time (constant or intermittent). Unilateral and constant nasal obstruction may originate as nasal septum deviation, foreign bodies, tumors, polyps, bilateral choanal imperforations and septal abscesses (Klein, 1987)^17^. However, the mouth breathing and malocclusion development relationship is controversial because some authors do not associate nasal obstruction as a primary factor causing malocclusion, but rather a factor of muscle unbalance between internal muscles (tongue more antero-inferiorly positioned) and pressure excess of cheek muscles against the maxilla, leaving the upper arch in a “V” shape in large adenoid patients, which would impact the orthodontic treatment stability (Quick & Gundlach (1978)^18^, Diamond (1980)^19^, Subtelny (1980)^20^, Vig et al. (1981)^21^, O’Ryan et al., (1982)^22^, Bressolin et al. (1984), Santos-Pinto & Monnerat (1986)^23^, Klein (1986)^24^, Meredith (1988)^25^, Cooper (1989)^1^, Smith & Gonzales (1989)^26^, Tourne (1990)^27^, Fields et al. (1991)^28^, Woodside et al. (1991), Vig (1998), Castilho et al. (2002)^29^, Solow & Greve, 1980)^30^.

Authors like: Watson et al. (1968), Muñoz (1970), Quick & Gundlach (1978)^18^, Diamond (1980)^19^, Subtelny (1980)^20^, Vig et al. (1981)^21^, O’Ryan et al., (1982)^22^, Bressolin et al. (1984), Santos-Pinto & Monnerat (1986)^23^, Klein (1986)^24^, Meredith (1988)^25^, Cooper (1989)^1^, Smith & Gonzales (1989)^26^, Tourne (1990)^27^, Fields et al. (1991)^28^, Woodside et al. (1991), Vig (1998), Castilho et al. (2002)^29^, did not find direct evidences of this relationship between respiratory patterns and malocclusions.

For mouth breathers, many studies associate head and neck position with body posture. Extended head position causes changes in many mobile anatomical elements between the head and neck, such as increasing the distance between the occipital and the dorsal arch of the first cervical vertebrae. This position rotates the head upwards, facilitating the passage of air through mouth and pharynx, thus facilitating muscle and skeletal system adaptations by postural and functional alterations also from the lips, tongue, masticatory muscles, mandible, soft palate and ocular muscles (Rahal & Krakauer, 2001, Harvold et al. (1973)^31^, Koski & Lähdemäki (1975), Rubin (1980), Harvold et al. (1981)^32^, Weber et al. (1981)^33^, Miller et al. (1984)^34^, Tarvonen & Koski (1987), Tourne (1990)^27^, Jabur et al. (1997), Para Mocellin & Ciuffi (1997), Jorge (2001)^35^, Simas Netta et al. (2004)^15^.

In a literature review, O’Ryan et al. (1982)^22^ studied the relationship between respiratory function and dento-facial morphology. Although many papers suggest a direct cause an effect relationship between nasal airway obstruction and dento-facial alterations, the authors concluded that there is a need for studies to quantitatively and longitudinally assess nasal and oral air flow during breathing, before we conclude that the respiratory obstruction is responsible for the development of a specific dento-facial disfigurement.

A direct cause and effect relationship between nasal and mouth breathing obstruction and altered dento-facial morphology and a precise diagnosis of the breathing pattern are necessary for the airway obstruction to be indicated as a significant etiological factor responsible for some specific dento-facial deformity, Schulhof (1978), Ianni Filho et al. (2001)^36^.

Nasal respiratory function studies should be objective, using accurate tests to asses the respiratory mode. Some use clinical interviews and physical exams, like Massler & Zwemer (1953)^37^, and clinical tests, like Quinn (1983)^38^. Others advocate the use of a cotton ball placed underneath the nose and/or mirrors placed in an alternate way in front of the nose during breathing. Cephalometrics are used to analyze the degree of oropharynx and nasopharynx obstruction, like McNamara Jr. (1984).

Rhinomanometry, together with an interview and clinical exams is also used to quantify nasal respiratory resistance. (Thuer et al., 1989)^39^.

Videoendoscopy has been considered a revolutionary diagnostic method, because with the use of flexible or rigid telescopes we can have a direct viewing of the pharyngeal tonsils, the nasal cavity, palatine tonsils, and the very size of the free space on the nasopharynx region. As to a proper assessment of the real degree of nasal obstruction through techniques that correspond to reality and allow proper therapy, nasofibroscopy has proven to be superior to lateral x-ray of the nasopharynx in assessing nasopharyngeal obstruction (Chami, 1998)^40^.

Having in mind the importance of studying exclusive oral breathers and its possible consequences for the craniofacial complex, our present study aimed at making cephalometric comparisons of young people with different respiratory patterns, observing the following aspects:

a) craniofacial growth pattern;
b) facial profile;
c) the relationship of apical bases with the skull;
d) anterior and posterior facial heights, and facial height index;
e) Check whether or not there are differences between nasal breathers and mouth breathers.

## MATERIALS AND METHODS

### Sample selection

This work only started after being approved by the ethics committee for research with human beings (CEP) - FOP-UNICAMP, according to documentation required by resolution 196/96 of the National Committee of Research Ethics (CONEP) from the National Health Council – Ministry of Health. The material used in the present study was made up of teleradiographies from the archival of orthodontic documentation from the Orthodontics Department – Children Orthodontics - FOP/UNICAMP, where 50 teleradiographies were selected, taken in lateral normal and natural head position of 50 Brazilian, white, female children, from 9 to 12 years of age, from first grade schools of the Limeira City Public School District.

We also used the otolaryngologist report, and divided the sample into control group, nasal breathers (n = 25) and experimental group: exclusive oral breathers (n = 25).

### Criteria for sample acquisition and selection

**Chart 1:**
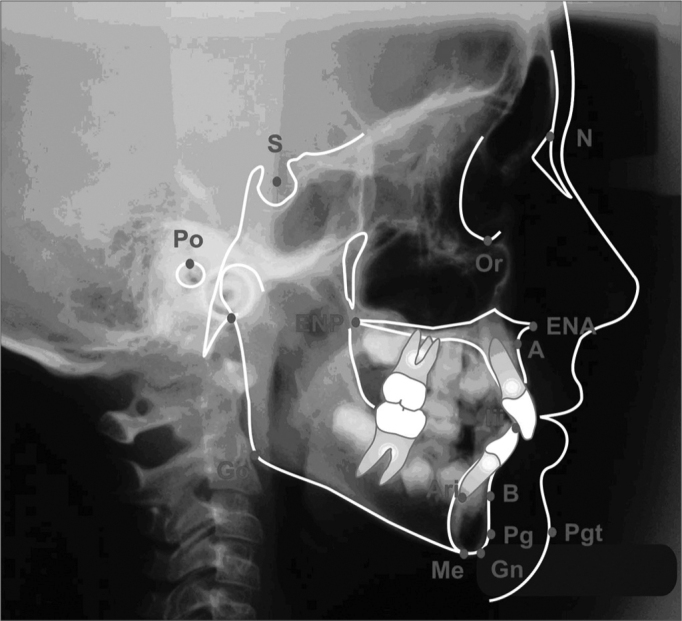
Adopted criteria for sample selection.

For sample selection for the present study we followed the criteria present on Chart 1:

### Nasofibroscopy exam

All children underwent a previous assessment by the otolaryngologist, responsible for the respiratory pattern diagnosis. Throat and nose were examined through clinical exam and nasofibroscopy. The otolaryngologist evaluated the questionnaire answered by the parents, history taken by the researcher, the normal-lateral and natural head position views, of which elements participated on the respiratory pattern diagnosis, classifying them as clinically normal respiratory pattern or as exclusive oral breathers.

The otolaryngologist used the respiratory pattern diagnostic protocol based on works by Wang et al. (1997), Ianni Filho (2001)^36^ and video-endoscopic results in order to issue the final report for each child respiratory pattern. The respiratory pattern diagnostic report and the videocassette recordings of the nasofibroscopy became part of the archrivals of Orthodontics from the Department of Children Dentistry - FOP/UNICAMP.

### Radiographic Method

Teleradiographies were taken in normo-lateral and natural head position views, always by the same operator – technician from the Dental Documentation Department, following the guidelines established by the School of Dentistry of Piracicaba - UNICAMP.

According to patients database data, in order to select the sample, the children were instructed to keep their teeth in habitual occlusion, lips relaxed and be comfortable in the upright position, looking at a mirror located 1m away. Before properly positioning the patient in the cephalostate, the head and body positions were practiced and repeated if necessary, in such a way that the pupils would be in the center of the eye.

The probes were then placed in the external auditory meatus, causing minimal pressure.

Once the patient confirmed face to face head position, the nasal support was placed on the nasion, lightly touching the head, just to establish vertical support, preventing any head movement.

The children were then instructed to keep their teeth in habitual occlusion and lips relaxed. To avoid a false impression of the nasopharynx obstruction, the children were asked to swallow before taking the x-ray where the patient's right side came closer to the radiographic film.

Methods were used to protect both the patient and the operator, such as lead apron to absorb secondary radiation.

The device used was Radiograph Plus X-Rays, manufacturer: Villa Sistemi Medicali & r.l. (Italy), which has total filtering equivalent to 25mm and focus size of 0.6mm ´0.6mm.

It was calibrated to operate with 16mA e 77KV, with a 0.4 to 0.5 exposure time, depending on the child's body mass.

The film used was T-MAT G/RA-1(Kodak Brasileira Com. e Ind. Ltda. -S.J. Campos, SP, Brasil), size 18 cm / 24 cm and the ecran was Lanex X-OMATIC Médium.

For development we used Revel (X-Tec-processadora de Raio X Ltda. Me, Brasil) –automatic processor, with augmented developer and fixator RP X-OMATI (Kodak Brasileira Com. e Ind. Ltda., S.J. Campos, SP, Brasil).

In order to show facial profile soft tissue, we used aluminum filter, positioned close to the radiation beam collimator.

The presented distortion coefficient was 10% in average.

Merrifield & Klontz (1993), skeletal figures were used in the cephalometric analysis, and included in the SN-GoGnand Y axis angles measurements. Therefore, three linear and eight angular variables were assessed. Two cephalograms were taken for each teleradiography, by the same investigator in a dark room and over the negatoscope in the Dentistry School of Piracicaba/UNICAMP, not following the numeric order established initially for patients and re-evaluated within a one week interval. The values were determined for the averages of these values.

### Cephalometric evaluation

**Figure 1:**
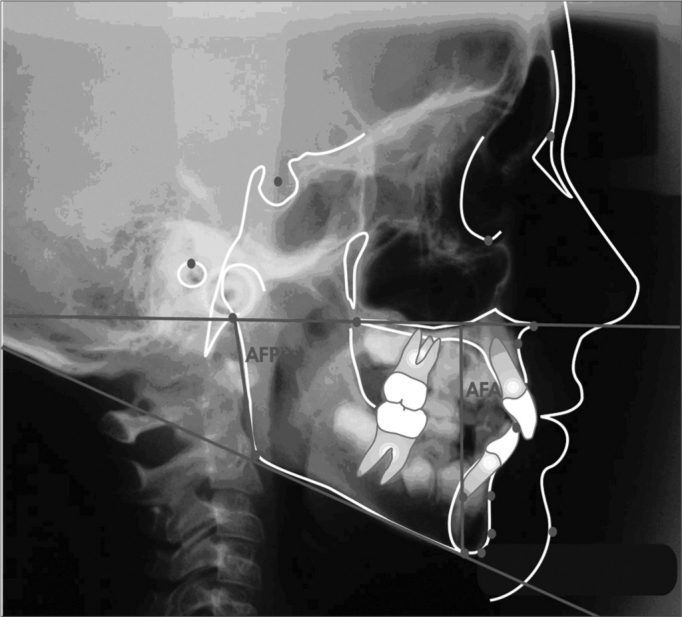
Cephalometric points

Anatomical structures outlining was carried out according to the orthodontics course tracing protocol from FOP/Unicamp, (according to Krogman & Sassouni, 1957; Interlandi, 1968; Vion, 1994), and we considered the smaller image, corresponding to the films closest side, with less distortion. The works from Steiner (1953), Krogman & Sassouni (1957)^41^, Interlandi (1968) and Horn (1992), as seen on Figure 1, were used to outline the cephalometric points and establishing the guiding traces

a) Saddle (S) – Turkish saddle geometric center, set by inspection;
b) Nasion (N) – nasal suture intersection with naso-frontal suture, in the median sagittal plane, set by inspection.
c) Porion (Po) – External acoustic meatus uppermost point. Very difficult to set because of other anatomical elements overlapping. To locate it, Miyashita (1996) reference points were used, in which the external acoustic meatus is located posteriorly to the mandible condillar process, above the basion and the axis odontoid process;
d) Orbitary (Or) – right orbit cavity contour lower most point;
e) Anterior nasal spine (ENA) – median point formed by the extension of both maxillas in the anterior and inferior portion of the nasal floor;
f) Posterior nasal spine (ENP) – median point formed by the union of the posterior borders of both palatine bones;
g) A (Sub spinal) – anterior maxilla concaveness deepest point, between the anterior nasal spine and the upper dental arch alveolar limit;
h) B (Supramenton) – deepest point in the symphysis anterior concaveness;
i) Pogonion (Pg) – anterior most point in the mandibular symphysis;
j) Tegumentary pogonion (Pgt) – anterior most or most prominent point on the chin soft tissue, in the median sagittal plane;
k) Mentonian (Me) – lower most point in the mandibular symphysis contour;
l) Gnathion (Gn) – most anterior and inferior point in the mandibular symphysis;
m) Articular (Ar) – Intersection point on the external skull base contour with the mandible condillar process;
n) Gonion (Go) – most anterior and posterior mandible point in the antero-posterior direction. Located at the ramus posterior border, tangent with the mandible inferior border angle bisectrix;
o) Inferior root apex (Ari) – point in the lower most region of the lower central incisive root apex;
p) Lower incisive border (BII) – Lower central incisive crown uppermost border region point;

### Planes and lines outlining

**Figure 2:**
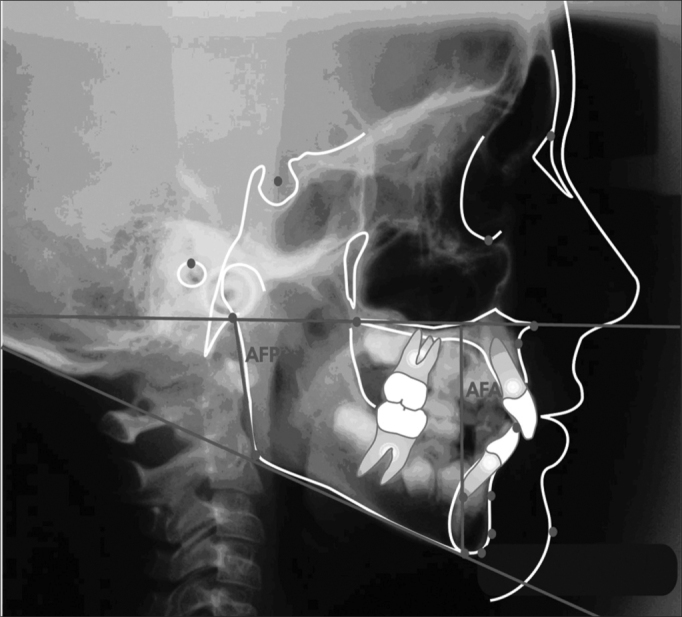
Cephalometric planes and lines outlining

After identifying the cephalometric points, planes and lines were traced, according to Figure 2.

**Table:** 

1-SN line	5- Go-Gn mandibular plane
2- Frankfut horizontal plane	6- Y axis
3- Palatine plane	7- NA Line
4- Mandibular plane Go-Me	8- NB
9- mandibular plane; inferior incisive long axis	10 line- Z line

### Measuring linear values

Posterior facial height (AFP) – Distance from the Ar point to the mandibular plane, in a tangence with the mandibular ramus posterior border (Merrifield, 1989).

Anterior facial height (AFA) – Distance between the Me point and its ortogonal projection over the palatine plane. The palatine plane is traced by joining ENP and ENA points (Merrifield, 1989).

Facial Height Index (IAF) – set by the AFP/AFA ratio (Horn, 1992).

### Measuring angular values

a) FMA – Angle formed by the intersection of the Frankfurt horizontal plane (PHF) with the mandibular (MD). The Frankfurt horizontal plane was traced by joining points Po and Or. The mandibular plane by the mandible body inferior border tangent, going through point Me (Tweed; 1954).
b) SNA – Angle formed by the intersection of the Saddle-Nasion and Nasion-A lines (Riedel; 1952).
c) SNB – Angle formed by the intersection of the Saddle-Nasion and Nasion-B lines (Riedel; 1952).
d) ANB – Angle formed by the intersection of the Nasion-A and Nasion-B lines (Riedel; 1952).
e) Angle Z – Formed by the intersection of the Frankfurt horizontal plane with the Pg point tangent line and the most anterior lip (Merrifield; 1966).
f) Y axis angle – Formed by the Y axis, from point (S) to point Gn, with the Frankfurt horizontal plane (Downs; 1948).
g) SN-GoGn Angle – Formed by the intersection of the mandibular plane (GoGn) with the S-N line.
h) IMPA angle – mandibular plane intersection with the lower central incisive long axis.

### Statistical analysis

This research statistical planning was carried out in two stages: first we calculated the error, which determines the intra-examiner error degree made during two moments, aiming at achieving greater reliability in the totality of traces and measures attained.

Second stage corresponds to the statistical analysis used to asses angular and linear cephalometric values used in the present study.

### Error calculation

In order to asses the method error in attaining the cephalometric values used in this study, in a way as to treat the data attained, and thus increase their accuracy and reliability, the same cephalograms were totally repeated after 30 days.

Error calculation was determined according to Dalberg's formula and advocated by Houston, in 1983, as follows;

where d is the repeatability deviation standard and: di is the individual error, i is the average error, and n is the number of individuals. We also carried out a paired “t” test with a 5% significance level comparing both traces. In an attempt to refine the attained data and increase their accuracy and reliability, each teleradiography was traced twice by the same investigator, thus yielding two values for each cephalometric variable. Calculating the simple arithmetic average we achieved the average value, and it was used in calculating skeletal values in the cephalometric analysis, from Merrifield & Klontz (1993), including SN-GoGn and Y angles. Therefore, two linear, one percentage and eight angular variables were analyzed.

In order to analyze the data, first we obtained a descriptive analysis (average and standard deviation) and later we applied the “t” Student test with 5% significance level.

## RESULTS AND DISCUSSION

**Figure 6:**
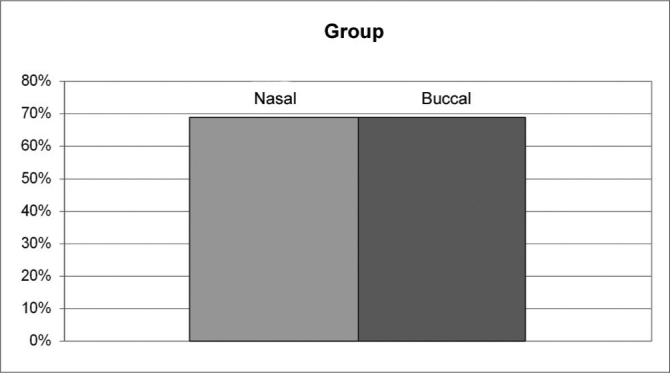
Linear values average and standard deviations for the predominantly nasal and oral groups.

**Figure 7:**
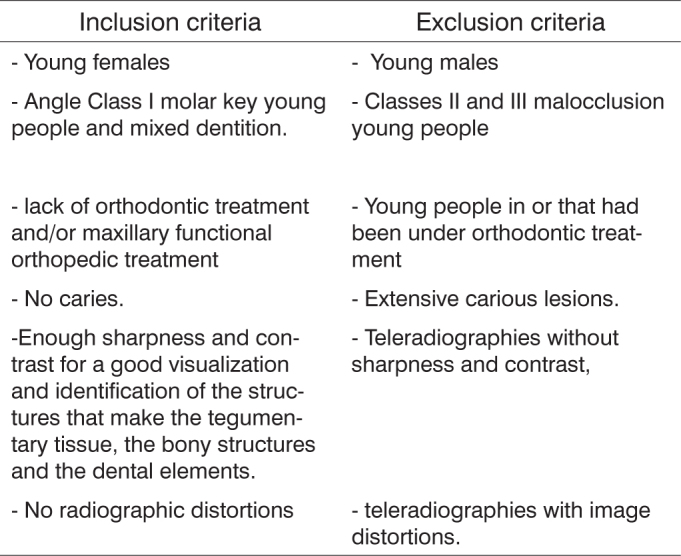
Average and standard deviation of the percent value, facial height index (IAF) for predominantly nasal and buccal groups.

Table 1 shows the error calculation for the duplicated measures in the nasal group. We did not see statistically significant difference between the traces (p > 0.05), thus attaining data reliability. Table 3 shows the average and standard deviation for values and the error, as well as the “t” test, comparing the traces at two moments for both groups. Table 4 shows the averages of 11 cephalometric values attained after evaluating 50 teleradiographies. There were no significant differences between the control group (nasal breathing) and the experimental group (predominantly oral breathers), p > 0.05. Figures 5, 6 and 7 depict the angle, linear and percent variation graphs respectively for both groups studied.

**Table 1:** Average, standard deviation and t test comparing outlines at both moments for the nasal group.

Values	Moment 1		Moment 2		Error		T Test
	Average	Standard Deviation	Average	Standard Deviation	Average	Standard Deviation	
IMPA	99,52	5,13	99,12	4,99		1,61	0,39
SNA	83,00	3,30	82,88	3,70		0,55	0,45
SNB	80,06	3,14	79,82	3,36		0,92	0,37
ANB	2,98	1,21	2,88	1,28		0,71	0,62
FMA	25,04	3,42	24,96	3,19		0,73	0,70
AFP	41,48	2,54	41,56	2,58		0,40	0,49
AFA	59,08	3,43	58,96	3,72		1,14	0,71
IAF	69,74	4,40	69,72	4,40		1,49	0,96
Z	68,40	5,58	67,68	5,35		1,94	0,20
SN-GoGn	33,36	4,03	33,70	3,89		0,77	0,13
Y	56,64	2,45	56,64	2,64		0,89	1,00

**Figure 3:**
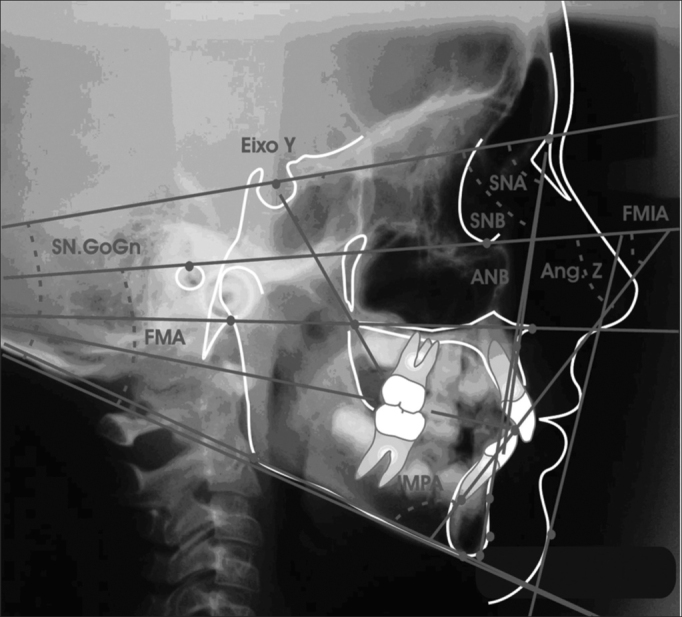
Linear values AFP – posterior facial height; AFA – anterior facial height

**Figure 4:**
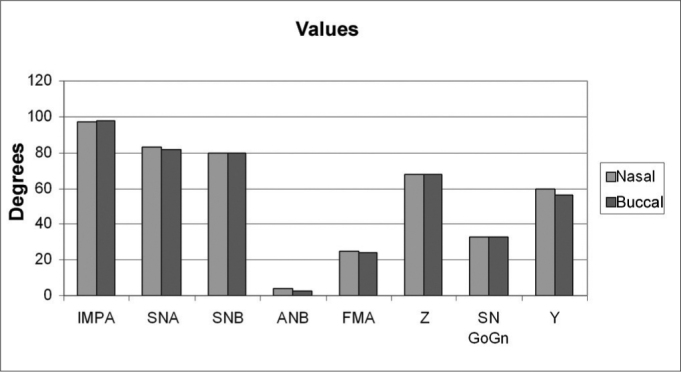
Angular values

**Figure 5:**
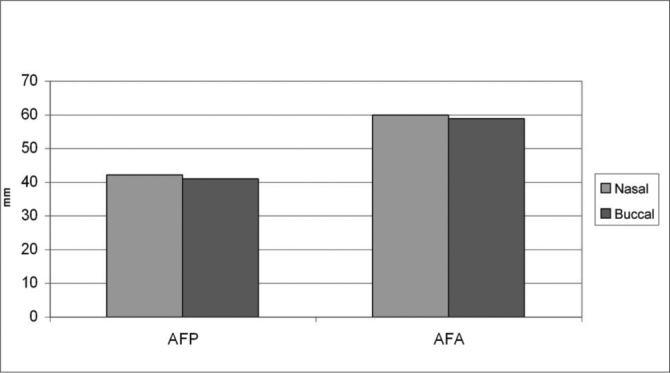
Angular values average and standard deviations for the predominantly nasal and oral groups.

The respiratory function and occlusion development relationship is a controversial subject. Authors such as Ricketts (1968), Cooper (1989)^1^, Behlfelt et al. (1990), Fields et al. (1991)^28^, Mocellin (1992) and Fujiki & Rossato (1999)^11^ directly appointed oral breathing as a malocclusion primary factor. Other authors such as Emslie et al. (1952), Rubin (1980), Subtelny (1980)^20^, Harvold et al. (1981)^32^, McNamara Jr. (1981)7, Weber et al. (1981)^33^, Linder-Aronson & Woodside (1982), Miller et al. (1982)^42^, Tomer & Harvold (1982), Solow et al. (1984)^43^, Cheng et al. (1988) and Jabur (1997) appointed oral breathing as a neuromuscular unbalancing factor that secondarily could cause or even increase malocclusion. Literary criticism as to the results found in predominantly oral breathers is that most of the times the respiratory pattern is diagnosed without scientific basis. Authors like Emslie et al. (1952), Massler & Zwemer (1953)^37^, Diamond (1980)^19^, Vig et al. (1981)^21^, Klein (1986)^24^, Cheng et al. (1988), Cooper (1989)^1^, Smith & Gonzales (1989)^26^, Vig (1998), Crouse et al. (2000)^44^, Parolo & Bianchini (2000), Queluz & Gimenez (2000)^2^, Ianni Filho et al. (2001)^36^ and Jorge (2001)^35^ concluded that oral breathing may not be subjectively diagnosed. Papers such as the ones from Miller et al. (1982)^42^ and Tourne (1990)^27^ call our attention to the studies carried out in human beings in which the oropharynx anatomy and monkey muscles data show that there is a need to exert great care in extrapolating data from experiments with these animals to the human population.

Our goal was to use cephalometrics to compare nasal breathers with predominantly oral breathers. In this context, following the hypothesis formulated, we may notice, by the results found, that there are no significant alterations, determined by the “t” test, at 5%, for any of the measures studied, when comparing nasal breathers with oral breathers.

Thus, when we analyze craniofacial growth patterns, determined by FMA, SN-GoGn and Y angle, values we may see, through the achieved results, a balanced pattern for nasal breathers as well as for oral breathers, in other words, in the nasal breathers group the head FMA assessed in PNC presented an average value of 25.44 ± 6.66, showing a mesofacial pattern, although with great variability shown by the high standard deviation value. On the other hand, when compared to oral breathers 24.96 ± 3.27 “t” test proven statistically significant differences were not seen between the two groups assessed. As for the SN-GoGn value, nasal breathers presented an average value of 33.4 ± 4.39, showing a mesofacial pattern with a significant variability shown by the standard deviation. On the other hand, when compared to oral breathers 33.79 ± 4.57 “t” student test proven statistically significant alterations were not seen between the two groups assessed. The Y angle in nasal breathers presented an average value of 59.16 ± 3.25 and 58.92 ± 3.37 for predominantly oral breathers, showing a mesofacial pattern also with significant variability shown by the standard deviation. For this variable, when both groups are compared, “t” student test proven statistically significant alterations were not seen. The results of this study did not indicate any facial pattern difference when both groups were compared, disagreeing from the results by the following authors: Koski & Lähdemäki (1975), Schulhof (1978), Rubin (1980), Harvold et al. (1981)^32^, McNamara Jr (1981)^7^, Bresolin et al. (1983)^8^, Bressolin et al. (1984), Miller et al. (1984)^34^, Solow et al. (1984)^43^, Santos-Pinto & Monnerat (1986)^23^, Melsen et al. (1987)^9^, Cheng et al. (1988), Martins (1988), Jabur et al. (1997), Yamada et al. (1997)^10^, Fujiki & Rossato (1999)^11^, Motonaga et al. (2000)^14^ and Pereira et al. (2001); and corroborating with the results of authors Linder-Aronson & Bäckström (1960), Quick & Gundlach (1978)^18^, Tarvonen & Koski (1987), Smith & Gonzales (1989)^26^, Bizetto (2000)^13^ and Mello (2001).

As to the Z-angle determined facial profile we can see a reduced value for both groups of nasal and oral breathers. Nasal breathers presented an average value of 67.90 ± 8.55, suggesting a convex profile, with significant variability, shown by a high standard deviation. On the other hand, when compared to oral breathers 68.4 ± 5.29, also suggesting a convex profile, “t” student test proven statistically significant alterations were not observed between the two groups assessed.

Altered facial profile may be related to a lower incisive protrusion, that may be noticed by higher value results found for both oral and nasal breathers. Nasal breathers had an average value of 98.32 ± 4.57, which is higher than the so considered normal average, and a significant variability shown by a high standard deviation value. Notwithstanding, when compared to oral breathers 99.26 ± 4.82, also suggesting oral dominance, “t” student test proven statistically significant alterations were not observed. Results found for this value corroborate Smith & Gonzales (1989)^26^ and differ from the results of authors like Hawkins (1969)^5^, McNamara Jr (1981)^7^, Bresolin et al. (1983)^8^, Bressolin et al. (1984), Santos-Pinto & Monnerat (1986)^23^, Cheng et al. (1988), Behlfelt et al. (1990), Fields et al. (1991)^28^, Mocelin (1992)^4^, Marchesan et al. (1995), Mocellin & Ciuffi (1997), Fujiki & Rossato (1999)^11^, Motonaga et al. (2000)^14^, and Pereira et al. (2001), who found a more convex profile in oral breathers when compared to nasal breathers.

As to bone base relations with the skull base, assessed by SNA, SNB and ANB values respectively, we observed that the results found for nasal breathers, SNA angle of 83.54 ± 2.91, showed a slightly higher value, however still within the normality pattern variation (82). For oral breathers, SNA angle showed an average value of 82.84 ± 3.46, also within normal standard variation. When we compared SNA angle from nasal breathers with that from oral breathers we did not find “t” student proven statistically significant alterations.

The SNB angle value was of 79.86 ± 3.12 for nasal breathers, and this is within the normal standard variation (80). For predominantly oral breathers, the SNB angle presented an average value of 79.96 ± 3.20, also within the normal standard variation. On the other hand, when comparing nasal breathers with oral breathers we did not observe “t” student proven statistically significant alterations.

The value attained for ANB was 3.49 ± 1.02, for nasal breathers, thus classifying the group as skeletal Class I. For oral breathers, the ANB presented an average value of 2.98 ± 1.21, also within class I variation pattern. On the other hand, when we compared nasal breathers with oral breathers we did not observe “t” student proven statistically significant alterations. The results found in the present study are contrary to those found by Hawkins (1969)^5^, Paul & Nanda (1973), Harvold et al. (1981)^32^, Bressolin et al. (1984), Santos-Pinto & Monnerat (1986)^23^, Melsen et al. (1987)^9^, who found an increase in ANB, not characterizing class I pattern (ANB varying from 1 to 5). Notwithstanding, the results found in the present study corroborate those from authors Linder-Aronson & Bäckström (1960), Watson et al. (1968), Muñoz (1970), Carbone & Bernaba (1977), Schulhof (1978) and Smith & Gonzales (1989)^26^ who also did not find any statistically significant difference in bone base relationships.

For posterior facial height, determined by AFP linear value, based on the results found, we see a reduced value for both oral and nasal breathers. Nasal breathers presented an average value of 41.0 ± 3.6, suggesting a lack of ramus height growth, with variability average shown by the standard deviation. In oral breathers, AFT presented a value of 41.66 ± 2.88, also suggesting a lack of ramus height growth, with average variability shown by the standard deviation. On the other hand, when compared between themselves, ‘t” Student proven statistically significant alterations were not seen between the groups.

Anterior facial height, determined by the AFA linear value, presented a reduced value for both groups. Nasal breathers presented average value 59.72 ± 3.97, suggesting a reduction in the vertical distance between the palatine plane and the mentum, with high variability shown by the standard deviation value. Within the oral breathers group, AFA presented a value of 59.84 ± 4.00, also suggesting a reduction in the vertical distance between the palatine plane and the mentum, with high variability shown by the standard deviation. O the other hand, when compared to each other, “t” Student proven statistically significant alterations were not observed between the two groups.

Facial height index, IAF determined, shows an AFP/AFA ratio within the balanced variation for both groups, despite a high variability shown by a high standard deviation, with a value of 69.72 ± 6.45 for oral breathers and 69.36 ± 6. for nasal breathers (normality IAF pattern = 69%). IAF between both groups, when compared to each other, did not show “t” Student proven statistically significant alterations. The results attained in this study corroborate those from authors Quick & Gundlach (1978)^18^, Linder-Aronson (1979)^6^ e Smith & Gonzales (1989)^26^ and are contrary to results from Harvold et al. (1973)^31^, Linder-Aronson (1979)^6^, Bressolin et al. (1984), Santos-Pinto & Monnerat (1986)^23^, Melsen et al. (1987)^9^ and Martinez Esteinou & Omana Vidal (1988), who found an increase in the anterior facial height, also leading to an alteration in the facial height index.

We have noticed that there were no significant alterations between the two groups when we compared the values achieved in the present study, thus confirming the concern that oral breathing may not be considered a primary factor in malocclusion for this sample studied.

## CONCLUSIONS

Based on these results, we may conclude that:

a) There was no statistically significant difference when facial patterns were assessed for both groups (mesofacial), represented by the values: FMA, SN-GoGn and Y axis angle;
b) Considering facial profiles in the sample studied, characterized by the Z angle, both groups had statistically similar behavior (convex profile);
c) As to the apical bases relationship, represented by values SNA, SNB, ANB, we may notice that there were no statistically significant alterations, as well as for anterior and posterior facial heights.
d) Among nasal and oral breathers we did not see any proof of statistically significant differences among the values considered, therefore, based on our results we have concluded that oral breathing may not always be considered as the single etiological factor responsible for facial pattern changes.
e) Since our study bears transversal characteristics, we suggest new longitudinal studies.
